# Pretreatment with Pancaspase Inhibitor (Z-VAD-FMK) Delays but Does Not Prevent Intraperitoneal Heat-Killed Group B Streptococcus-Induced Preterm Delivery in a Pregnant Mouse Model

**DOI:** 10.1155/2009/749432

**Published:** 2009-12-28

**Authors:** Ozlem Equils, Chantelle Moffatt-Blue, Tomo-o Ishikawa, Charles F. Simmons, Vladimir Ilievski, Emmet Hirsch

**Affiliations:** ^1^Division of Pediatric Infectious Diseases, Steven Spielberg Pediatric Research Center, Burns and Allen Research Institute, Cedars-Sinai Medical Center, Los Angeles, CA 90048, USA; ^2^Ahmanson Department of Pediatrics, Steven Spielberg Pediatric Research Center, Burns and Allen Research Institute, Cedars-Sinai Medical Center, Los Angeles, CA 90048, USA; ^3^Departments of Obstetrics and Gynecology, David Geffen School of Medicine at UCLA, Evanston, IL, USA; ^4^Medical Division, Pfizer Inc., New York, NY 10017-5755, USA; ^5^NorthShore University HealthSystem, Evanston, IL 60201, USA; ^6^Pritzker School of Medicine, University of Chicago, Chicago, IL 60637, USA

## Abstract

Caspases and apoptosis are thought to play a role in infection-associated preterm-delivery. We have shown that in vitro treatment with pancaspase inhibitor Z-VAD-FMK protects trophoblasts from microbial antigen-induced apoptosis. *Objective*. To examine whether in vivo administration of Z-VAD-FMK would prevent infection-induced preterm-delivery. *Methods*. We injected 14.5 day-pregnant-mice with heat-killed group B streptococcus (HK-GBS). Apoptosis within placentas and membranes was assessed by TUNEL staining. Calpain expression and caspase-3 activation were assessed by immunohistochemistry. Preterm-delivery was defined as expulsion of a fetus within 48 hours after injection. *Results*. Intrauterine (i.u.) or intraperitoneal (i.p.) HK-GBS injection led to preterm-delivery and induced apoptosis in placentas and membranes at 14 hours. The expression of calpain, a caspase-independent inducer of apoptosis, was increased in placenta. Treatment with the specific caspase inhibitor Z-VAD-FMK (i.p.) prior to HK-GBS (i.p.) delayed but did not prevent preterm-delivery. *Conclusion*. Caspase-dependent apoptosis appears to play a role in the timing but not the occurrence of GBS-induced preterm delivery in the mouse.

## 1. Introduction

Preterm birth is the most common cause of death in newborn babies worldwide [1–3]. In the US preterm delivery is one of the most significant complications of pregnancy. Approximately 34% of infant mortality is due to preterm delivery in the US [4]. It has a high prevalence rate (11%), and about 40% (> $4 billion) of all infant health care expenditures in the US are related to prematurity [5].

Infection is the most common cause of preterm delivery and stillbirth globally. In the US infection plays a role in approximately 50% of total and 80% of early preterm deliveries (<32 weeks of gestation) [6, 7]. However, despite being one of the most important maternal-fetal problems, there are no effective prevention strategies or treatments for infection-induced preterm delivery, and there is no thorough understanding of the molecular mechanisms involved.

Thus far studies investigating the mechanisms involved in infection associated preterm delivery have concentrated on inflammatory signaling pathways [8]. Yet, in vivo and in vitro human and animal pregnancy data suggest that infection can also induce apoptosis in the placenta and the membranes [9–23].

Most recently, caspases were shown to be activated upon microbial antigen treatment of human trophoblasts [16, 17]. We have shown that in vitro pretreatment of primary human trophoblasts and placental fibroblasts with pancaspase inhibitor Z-VAD-FMK prevented chlamydia heat shock protein 60-induced apoptosis [17].

Group B streptococcus is one of the most common causes of neonatal infection and is associated with preterm delivery [24]. Here we show that both intrauterine (i.u.) and intraperitoneal treatment (i.p.) with heat-killed Group B streptococcus (HK-GBS) induce preterm delivery in day 14.5 pregnant mice. We next tested whether pretreatment with the pancaspase inhibitor Z-VAD-FMK prevents HK-GBS-induced preterm delivery in vivo.

## 2. Materials and Methods

### 2.1. Materials and Reagents

Group B *β*-hemolytic streptococcus (GBS) bacteria were grown to log phase at 37°C in Trypticase Soy Broth (Becton Dickinson), concentrated by centrifugation at 3000 G, resuspended in PBS, quantified by plating serial dilutions, and then heat-inactivated by boiling for 5 minutes. Bacterial killing was verified by lack of growth overnight in broth and solid media. Heat-killed (HK)-GBS stock was aliquoted and frozen at −80°C. Before each experiment, a fresh vial of frozen heat-killed bacteria was thawed, vortexed, diluted as necessary, and used in the experiments.

Cell-permeable Z-VAD-FMK (BD Pharmingen catalog number 550377) was dissolved in DMSO, aliquoted and stored at −80°C, and then diluted as needed in PBS for experiments. The final concentration of DMSO in the solution injected into the animal was less than 1%.

### 2.2. Model of Infection-Induced Preterm Delivery in Mice

The NorthShore University Health System Animal Care and Use Committee approved all animal procedures. A model of bacterially induced preterm delivery resulting from intrauterine inoculation has been described previously [25].

Briefly, timed-pregnant C57BL/6J mice (Jackson Laboratories, Bar Harbor, Maine) on day 14.5 of pregnancy were anesthetized with 0.015 ml/g body weight of 2.5% tribromoethyl alcohol and 2.5% tert-amyl alcohol in phosphate buffered saline (PBS). A 1.5 cm midline incision was made in the lower abdomen. The right uterine horn was identified and injected in its mid-section with either PBS or GBS (10^9^ organisms) in a 100 *μ*L volume delivered extraovularly between fetal sacs. The incision was closed with interrupted sutures of coated 4-0 polyglactin 910 sutures (Vicryl, Ethicon) at the peritoneum and wound clips at the skin. Surgical procedures lasted approximately 10 minutes. Animals were either observed through delivery or euthanized 5 or 14 hours after HK-GBS injection for tissue collection (placentas and membranes). These tissues were fixed in 10% neutral buffered formalin and embedded in paraffin for sectioning.

To assess whether pancaspase inhibitor Z-VAD-FMK prevents HK-GBS-induced preterm delivery, unanesthetized day 14.5 pregnant CD1 mice (Harlan Laboratories, Madison, WI), which breed more effectively than inbred C57BL/6J mice, were pretreated intraperitoneally with PBS, DMSO, or Z-VAD-FMK (10 mg/kg) 30 minutes prior to intraperitoneal injection with either 10^9^ HK-GBS bacteria or medium. Because there were no differences between the groups pretreated with either PBS or DMSO (diluents for the caspase inhibitor), these two groups were combined for the analyses.

Postoperatively, mice were observed for premature delivery (defined as the finding of at least one pup in the cage or the lower vagina within 48 hours of the intervention, as previously described [25]).

### 2.3. TUNEL Staining

Apoptosis was assessed by the in situ terminal deoxynucleotidyl transferase- (TdT-) mediated dUTP nick end-labeling (TUNEL) technique with the TACS 2TdT Blue Label kit (Trevigen, Gaithersburg, MD, USA) according to the protocol supplied by the manufacturer. Positive control sections were pretreated with TACS-Nuclease to induce DNA fragmentation before the TUNEL reaction. Negative controls were processed in the absence of the TdT enzyme and showed no staining. Mouse ovaries were used as positive control tissues.

### 2.4. Immunohistochemistry

Paraffin-embedded tissue sections were deparaffinized in xylene, rehydrated through a series of ethanol solutions, and then rinsed in PBS. Sections were placed in Antigen Unmasking Solution (Vector Laboratories, Burlingame, CA, USA) and heated in a pressure cooker for 10 minutes. Endogenous peroxidases were quenched in a 3% H_2_O_2_/methanol solution. After 40 minutes of blocking with a 1 : 20 solution of normal goat serum to PBS/Tween 20, sections were incubated overnight at room temperature in a 1 : 350 dilution of an antibody to either activated caspase-3 (R&D Systems, Minneapolis, MN, USA) or m-calpain (GeneTex, Inc, San Antonio, TX). The sections were then rinsed in PBS and incubated for 45 minutes at room temperature with a biotinylated goat anti-rabbit immunoglobulin G secondary antibody (1 : 200; Vector Laboratories). Immediately after incubation with the secondary antibody, sections were incubated for 30 minutes at room temperature with avidin-biotin-peroxidase solution (Vectastain elite ABC kit; Vector Laboratories). The antigen was visualized with the NovaRed Substrate kit (Vector Laboratories) and counterstained with hematoxylin. Negative control sections were processed in the absence of the active caspase-3 primary antibody. Mouse ovarian sections containing atretic follicles were used as positive control tissues.

### 2.5. Statistical Analysis

Fisher's exact test was performed to assess the effect of Z-VAD-FMK pretreatment on HK-GBS-induced preterm delivery.

## 3. Results

### 3.1. HK-GBS Injection Induces Preterm Delivery in Pregnant Mice

Intraperitoneal inoculation with heat-killed GBS (10^9^) in day 14.5 pregnant mice induced preterm delivery (Table 1). No mother died during the course of the experiment. Similar results were obtained in animals exposed to intrauterine HK-GBS (data not shown). These data confirm that HK-GBS exposure leads to preterm delivery in the mouse pregnancy model.

### 3.2. HK-GBS Injection Leads to Apoptosis in the Placenta and Membranes

**Figure 1:**
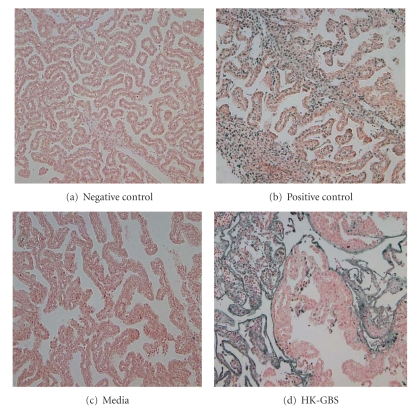
*Intrauterine HK-GBS injection leads to TUNEL positive apoptosis in the membranes*. Day 14.5 timed pregnant mice were injected with either HK-GBS (panel d) or PBS (a) and euthanized at 5 or 14 hours to isolate the placenta and membranes. The sections shown were obtained after 14 hours of stimulation. TUNEL positive apoptotic cells are stained black-brown. Slides treated with endonuclease served as a positive control (b); animals injected with media indicate baseline apoptosis levels (c). Data shown are representative of 3 separate experiments.

**Figure 2:**
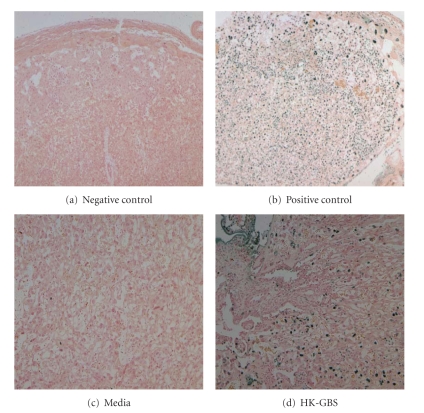
*Intrauterine HK-GBS injection leads to TUNEL-positive apoptosis in the placenta*. In the mouse placenta, there was TUNEL positive apoptosis after 14 hours of exposure to HK-GBS (d). The slides were treated with PBS for the negative control (a) and endonuclease for the positive control (b); animals were injected with media to assess baseline apoptosis levels (c). Data shown are the representative of 3 separate experiments.

The effect of HK-GBS exposure on placental and membrane apoptosis was assessed by TUNEL assay in day 14.5 pregnant mice euthanized at 5 (*n* = 4) or 14 hours (*n* = 6) after intrauterine bacterial injection. Apoptosis was detectable at membranes (Figure 1) and placentas (Figure 2) at 14 hours but not at 5 hours after bacterial exposure. 

**Figure 3:**
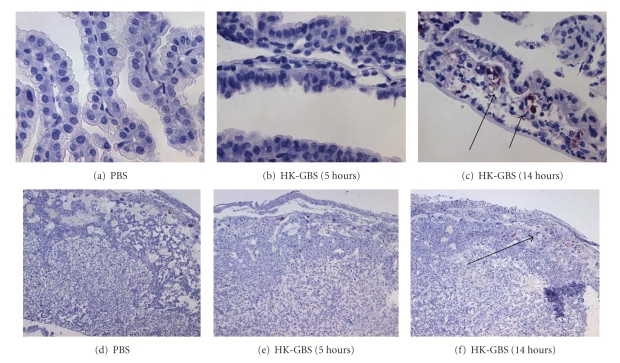
*Intrauterine HK-GBS injection leads to caspase 3 activation in the membranes and placenta*. Day 14.5 timed pregnant mice were injected with either PBS or HK-GBS and euthanized at 5 or 14 hours to isolate fetal membranes (a–c) and placentas (d–f). Caspase 3 activation was assessed by performing immunohistochemistry analysis using an antibody against active-cleaved caspase 3. Representative data from three separate experiments are shown. HK-GBS exposure led to an increase in caspase 3 positive cells in the membranes (c) and above the spongiform trophoblast layer at 14 hours in the placenta (f).

**Figure 4:**
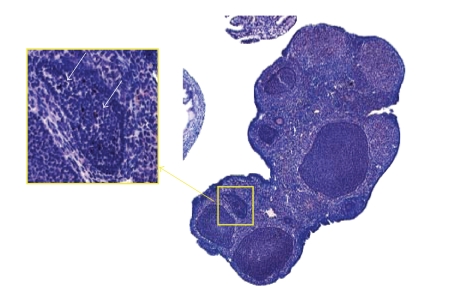
*Apoptosis in the ovarian follicle*. Mouse atretic follicles are known to undergo caspase mediated apoptosis. As anticipated, caspase 3 was cleaved and activated in the atretic follicle, which were used as positive control for the caspase 3 antibody specificity. Representative data from three separate experiments are shown.

Caspase 3 is the common executioner caspase activated by both the extrinsic (Fas) and intrinsic (mitochondrial) caspase machinery. Intrauterine HK-GBS exposure induced caspase 3 activation in a time-dependent manner (i.e., at 14 hours but not 5 hours) in the fetal membranes (Figures 3; 3(a)–3(c)) and in the placenta (Figures 3; 3(d)–3(f)) as assessed by immunohistochemistry using an antibody specific for activated cleaved caspase 3. In order to confirm the specificity of the caspase 3 staining, we used nonpregnant mouse ovaries as positive control tissue. As anticipated, caspase 3 was activated in the atretic ovarian follicles (Figure 4).

### 3.3. Pretreatment with Pancaspase Inhibitor Z-VAD-FMK Delays HK-GBS-Induced Preterm Delivery

We have previously shown that in vitro pretreatment with Z-VAD-FMK prevented Chlamydia heat shock protein- (cHSP60-) induced apoptosis in primary human trophoblasts and fibroblasts [17]. Based upon that observation, we hypothesized that in vivo treatment with Z-VAD-FMK would prevent microbial toxin-induced preterm delivery in the mouse pregnancy model. In order to test this hypothesis, we pretreated day 14.5 pregnant CD1 mice either with Z-VAD-FMK (10 mg/kg dissolved in DMSO/PBS) or with medium (DMSO/PBS) intraperitoneally 30 minutes prior to intraperitoneal HK-GBS injection. We observed the mothers closely for delivery within 48 hours.

Pretreatment with Z-VAD-FMK significantly delayed preterm delivery at 18 hours (Table 1; *P* = .007). However at 36 hours after treatment, there were no differences between the caspase inhibitor-pretreated and control groups (Table 1).

### 3.4. HK-GBS Induces Calpain Expression in the Placenta

GBS has been shown to induce macrophage apoptosis in a caspase-independent manner via m-calpains, which are calcium dependent cytosolic cysteine proteases [26]. Fettucciari et al. have shown that in vitro siRNA inhibition of calpain prevented GBS-induced apoptosis in macrophages, while caspase inhibition with Z-VAD-FMK did not [26].

**Figure 5:**
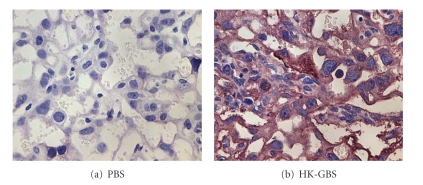
*Intrauterine HK-GBS injection induces placental m-calpain expression in a time-dependent manner*. Day 14.5 pregnant mice were euthanized 14 hours after exposure to PBS (i) or HK-GBS (ii). Placentas were removed and stained for m-calpain. Data from a representative of three separate experiments is shown.

We examined the effect of HK-GBS treatment on placental calpain expression and observed that i.u. HK-GBS injection led to a time-dependent increase in m-calpain expression in the mouse placenta within 14 hours of exposure as assessed by immunohistochemistry (Figure 5).

## 4. Discussion

Apoptosis is proposed to be a normal developmental process in the placenta and developing fetus and increases throughout gestation in humans [27, 28]. Apoptosis has been suggested to play a role in normal rupture of the membranes during labor [29]. In addition to its physiologic role in normal pregnancy and fetal development, there is accumulating data on the presence of apoptosis in pathologic pregnancies (i.e., preeclampsia [30], intrauterine growth restriction [31], and infection associated preterm delivery [13]).

Caspases are cysteine proteases with aspartate specificity and are the key mediators of apoptosis. Caspase 8 mediates the FAS-extrinsic pathway of caspase activation, whereas caspase 9 mediates intrinsic-mitochondrial caspase activation. Both caspase 8 and caspase 9 then activate caspase 3, which is one of the executioner caspases (reviewed in [32]). Infection leads to the expression of inflammatory cytokines such as TNF-*α*, IL-1, and IL-6 as well as other factors such as FasL, heat shock proteins, reactive oxygen species, and nitric oxide, all of which are known to regulate caspase activation [33–35]. In addition, infection can activate caspase 8 directly through the innate immune system via toll-like receptors (TLRs) and the adaptor molecule MyD88 [36–39]. MyD88 has a death domain and interacts with Fas-Associated protein with Death Domain (FADD) to activate caspase 8 [39]. 

In the present study, we showed that intraperitoneal injection with heat-killed GBS leads to preterm delivery and apoptosis in the placenta and membranes of 14.5 day pregnant mice. Next, we showed that HK-GBS exposure induces caspase 3 activation in the placenta and the membranes. These data confirm that apoptosis is a physiologic response to exposure to GBS in the reproductive tract during pregnancy.

*N*-benzyloxycarbonyl-Val-Ala-Asp-fluoromethylketone (Z-VAD-FMK) binds irreversibly to the catalytic site of caspases and inactivates them. In vivo Z-VAD-FMK administration has been shown previously to be nontoxic and to prevent apoptosis in animal models [40–42]. Here we treated pregnant animals with Z-VAD-FMK 30 minutes prior to intraperitoneal injection with HK-GBS and observed that Z-VAD-FMK treatment delayed, but did not prevent HK-GBS-induced preterm delivery.

HK-GBS may induce caspase-independent pathways as well as caspase-dependent ones [43]. Currently, there are no data on the role of caspase-independent apoptotic pathways in infection-associated preterm delivery. Similar to caspases, calpains are ubiquitously expressed cysteine proteases and play a role in caspase-independent apoptosis. In contrast to caspases, calpains are present only in the cytoplasm (e.g., caspase 9 is mitochondrial), and are regulated by intracellular Ca^2+^ level [44]. Calpains have been proposed to play a role in cancer, cardiovascular disease, Alzheimer's disease, multiple sclerosis, and polycystic ovary syndrome [45]. After binding to Ca^2+^ ions, calpains undergo a conformational change, which initiates proteolytic activity. Calpains then proteolyse a wide range of substrates including cytoskeletal components, plasma membrane-associated proteins such as epidermal growth-factor receptor and platelet derived growth-factor-receptor, and signal transduction and calmodulin-dependent proteins and transcription factors [44]. Shortly after substrate cleavage, calpains undergo autolytic cleavage that limits their enzymatic activity to a few minutes. Human placenta is abundant in calpains [46]. Although the role of calpains in the placenta is not clearly known, they were suggested to play a role in extravillous trophoblast migration [47]. Calpains have been shown to mediate GBS-induced caspase-independent apoptosis in macrophages [26]. 

The present finding that treatment with a caspase inhibitor delays GBS-induced preterm delivery suggests that apoptosis plays a role in bacterially-induced preterm labor. The fact that the delay in preterm delivery did not translate into a diminished overall rate of preterm birth within 48 hours can be explained by the existence of alternative or redundant pathways to caspase-dependent apoptosis that can lead preterm labor and delivery. Literature provides many examples of candidates for such alternate pathways, including cytokines, prostaglandins, and matrix metalloproteases [48–50]. Our finding that HK-GBS exposure induces m-calpain expression in the mouse placenta suggests the possibility that one such alternative pathway exists within the apoptotic mechanism itself. This observation may help explain the inability of Z-VAD-FMK to prevent HK-GBS-induced preterm delivery in our model. Alternatively Z-VAD-FMK treatment of the pregnant animals, at the dosages used, did not block caspase-3 activation which can be assessed by Tunnel assay or immunohistochemistry. Future experiments will explore the effect of treatment with different concentrations of Z-VAD-FMK and combined inhibition of caspase and calpain and their effects on preterm birth.

## 5. Conclusion

Here we show that intrauterine exposure to heat-killed GBS induces apoptosis in the placenta and membranes and leads to preterm delivery in the pregnant mouse model. HK-GBS treatment also leads to both caspase activation and calpain expression in the mouse placenta and membranes. Pretreatment of the pregnant animals with the pancaspase inhibitor Z-VAD-FMK delayed but did not prevent HK-GBS-induced preterm delivery. Our data suggest that exposure of placentas and membranes to microbial antigens leads to the induction of caspase-dependent and -independent apoptotic pathways.

## Figures and Tables

**Table 1:** *Intraperitoneal HK-GBS injection leads to preterm delivery, and pretreatment with Z-VAD-FMK delays preterm delivery in mice*. 10^9^ heat killed GBS bacteria were injected intraperitoneally in day 14.5 pregnant CD-1 mice. Data from animals treated with HK-GBS without Z-VAD-FMK (GBS alone, PBS + GBS or DMSO + GBS) were combined.

	Preterm delivery <18 hours (%)	Preterm delivery 18– <24 hours (%)	Preterm delivery 24–36 hours (%)
GBS (*n* = 14)	12 (86%)	0	2 (14%)
Z-VAD + GBS (*n* = 6)	1 (17%)	2 (33%)	3 (50%)
*P-*value	.0072	.079	.13
